# Performing post-genome-wide association study analysis: overview, challenges and recommendations

**DOI:** 10.12688/f1000research.53962.1

**Published:** 2021-10-04

**Authors:** Yagoub Adam, Chaimae Samtal, Jean-tristan Brandenburg, Oluwadamilare Falola, Ezekiel Adebiyi

**Affiliations:** 1 Covenant University Bioinformatics Research (CUBRe), Covenant University, Ota, Ogun, 112233, Nigeria; 2 Laboratory of Biotechnology, Environment, Agri-food and Health, Sidi Mohammed Ben Abdellah University, Fez, Fez-Meknes, 30000, Morocco; 3 Sydney Brenner Institute for Molecular Bioscience (SBIMB), University of the Witwatersrand, Johannesburg, South Africa; 4 Computer & Information Sciences, Covenant University, Ota, Ogun, 112233, Nigeria; 5 Covenant Applied Informatics and Communication Africa Centre of Excellence, Covenant University, Ota, Ogun, 112233, Nigeria; 6 Applied Bioinformatics Division, German Cancer Center DKFZ - Heidelberg University, Heidelberg, Baden-Württemberg, 69120, Germany

**Keywords:** PostGWAS, pGWAS, GWAS, Meta-analysis

## Abstract

Genome-wide association studies (GWAS) provide  huge information on statistically significant single-nucleotide polymorphisms (SNPs) associated with various human complex traits and diseases. By performing GWAS studies, scientists have successfully identified the association of hundreds of thousands to  millions of SNPs to a single phenotype. Moreover, the association of some SNPs with rare diseases has been intensively tested. However, classic GWAS studies have not yet provided solid, knowledgeable insight into functional and biological mechanisms underlying phenotypes or mechanisms of diseases. Therefore, several post-GWAS (pGWAS) methods have been recommended. Currently, there is no simple scientific document to provide a quick guide for performing pGWAS analysis. pGWAS is a crucial step for a better understanding of the biological machinery beyond the SNPs. Here, we provide an overview to performing pGWAS analysis and demonstrate the challenges behind each method. Furthermore, we direct readers to key articles for each pGWAS method and present the overall issues in pGWAS analysis.  Finally, we include a custom pGWAS pipeline to guide new users when performing their research.

## Introduction

Genome-wide association studies (GWAS) have been used to identify genetic variants associated with specific traits or diseases of interest. One of the advantages of performing GWAS is that they do not require prior knowledge of the biological hypothesis underpinning the genetic machinery of the investigated trait. Many GWAS have revealed hundreds of common variants that are associated with various phenotypes, including common diseases. ^1^ Edwards *et al* reported the first GWAS for age-related macular degeneration in 2005. ^2, 3^ In the last decade, many GWAS have been reported in scientific databases ^3^ and these studies have revealed the presence of various single nucleotide polymorphisms (SNPs). For instance, GWAS Catalog reported 4865 publications and 247051 associations in 2021. These reported SNPs could be used to better understand the molecular mechanisms of common diseases and biological pathways of interesting traits.

Over the last decade, most GWAS have been known to detect the genetic signals of a single gene or a single genetic marker, i.e., interpreting genes based on detected signals (positions) on genomic coordinates using a specified Genome Assembly. ^4^ However, most complex diseases that have been targeted by GWAS are known to be caused by multiple genes that could be influenced by many other factors. It is known that GWAS typically report SNPs as statistically significant when their associated *p*-values are less than 5e-08. Accordingly, GWAS might not detect genetic variants with low or moderate risk. ^5, 6^ Thus, the ability of GWAS to detect a significant genomic position depends on heritability on phenotype, minor allele frequency (MAF), and sample size. Traditional GWAS could therefore be faced with the challenge of not being able to detect variants that are associated with low disease risk, implying that traditional GWAS results are prone to unreliable findings due to their false negative results. ^7^ Furthermore, GWAS might fail to detect a significant signal if the effect of a variant in another gene is not taken into consideration. ^5^ It is also limited in identifying changes in genotype as a response to environmental changes, i.e., detecting the impact of genotype interaction with the environment. ^8^ These limitations of GWAS occur primarily because of the undetected effect of gene polymorphism. More specific challenges of GWAS have been reported in many scientific papers. ^1, 5, 7, 9- 12^ Given all of the reported limitations of GWAS, it is crucial to perform post-GWAS (pGWAS) analysis. The overall goal of pGWAS analysis is to use the result of the association between the genotype and phenotype (summary statistics), with the following objectives:

• Transferability of previous result,
• Identification of new significant functional variants, i.e. lead SNPs,
• Identification of novel disease susceptibility genes, genotype-phenotype associations, and biological pathway network, and
• Building a polygenic risk score using summary statistics.

So far, the most common approaches to perform pGWAS analysis include the following three approaches: (i) Single-variant approach; (ii) Gene-scoring approach; and (iii) Pathway-sub-network-based approach. However, pGWAS approaches could be categorized into many classes based on their usage (see section below). Also, there are several methods/tools for pGWAS reported in different articles.

In this article, we provide an overview to performing pGWAS analysis and discuss the challenges behind each method. Furthermore, we direct readers to key articles for each pGWAS method and present the overall issues in pGWAS analysis. Finally, we include a custom pGWAS pipeline to guide new users when performing their research.

## GWAS analysis

GWAS have become an indispensable approach in providing insight into understanding genotype-phenotype associations of complex disease. While the design for GWAS experiments are well established as bench-work, the computational methods for the analysis of GWAS data are still evolving. The typical computational pipeline to analyse any GWAS data consists of two essential tasks: a) upstream GWAS analysis (classic GWAS methods), and b) downstream GWAS analysis. The latter step is known as pGWAS analysis.

The upstream GWAS analysis is a multi-step task resulting in a list of statistically significant SNPs. This step often starts by checking the quality control of raw GWAS data which is a crucial step for the task of pGWAS analysis. It is obvious that unclean data lead to unreliable results. Therefore, many parameters such as quality control per sample, relatedness, replicate discordance, SNP quality control, sex inconsistencies, and chromosomal anomalies should be checked. ^13, 14^ After obtaining raw data-sets with high quality scores, the next step is to report the statistically significant SNPs. Many statistical tests are available for this, including Chi-square test, Fisher’s exact test, Cochran-Armitage trend test, Odds ratio, Logistic regression, ANOVA, Transmission Disequilibrium test, Bonferroni correction, and many other methods.

Many free tools are available to perform GWAS upstream data analysis such as: Plink, ^15^ PLATO, ^16^ EIGENSOFT, ^17^ and STRUCTURE. ^18^ Several other tools are also available as packages within R software, the most popular open-source software for statistical computing. ^19^

## pGWAS approaches

### Single-variant approach

**Genome wide significant threshold.** This approach provides the statistics at a SNP level. This approach aims to score the association between SNPs and the target trait. Usually, the score defined by a β value or Odd Ratio ( *OR*), and its standard error ( *SE*) gives an idea of the genotype’s effect on the phenotype. Effect strength is computed using *OR.* Also, scientists use *p*-value as a measure of how likely this effect is to occur by chance. Statistically, many researchers use *p*-value to evaluate the hypothesis that there is no statistical evidence for SNP-trait associations. However, this hypothesis will be rejected when *p*-value is less than a predetermined threshold that is adjusted for a multi-test. Many methods are used for multiple test *p*-value correction. These methods include Bonferroni correction and false discovery rate ( *FDR*). ^20^ However, some of these methods do not consider linkage disequilibrium ( *LD*) and they may be too stringent. In general, a threshold of 5e‐08 is acceptable for human association studies. ^21, 22^ This value has been computed using independent signals in genomes. However, this value is not absolute and it depends on many factors, including *LD* (and diversity), array type, whole genome sequencing or whole exome sequencing, position number in array or sequencing, imputation panels, and positions finally analyzed. ^23^ For trans-ethnic populations, it is recommended to estimate the threshold based on population diversity and *LD.* For instance, analyzing 1000 Genomes data, the suggested significance thresholds were 3.24e‐08 for Africa, 9.26e‐08 for Europe, 1.83e‐07 for Mixed America, 1.61e‐07 for East Asia and 9.46e‐08 for South Asia. ^24^ Although, a recent study using an African population proposed a threshold of 5e‐09 . ^25^

**Suggestive threshold.** Some authors consider GWA threshold very stringent, so studies often include a “suggestive threshold”. This is superior to a genome-wide significant threshold, and values found in the literature are 1e‐05 , 1e‐06 or lower. Studies estimated on “independant *LD* block” from 1000 Genomes used 1e‐05 for the Affymetrix 500K and Illumina 317K GWAS SNP panels, and 1e‐06 for HapMap CEPH Utah and Yoruba populations. ^26^ The code below contains an R command to select significant SNPs using a cutoff value of 5e‐08 .



## library need : data.table
#open files with fread function
data.gwas<-fread("result.gwas")
# head to obtain the header of gwas file
head(data.gwas)
# selected lines using threshold value of 5E-8
data.gwas[data.gwas$p.value<5*10**-8,]
# obtained information about min pvalue
data.gwas[which.min (data.gwas$p.value),]


**Inflation factors**. Population and cryptic relatedness can cause spurious associations in GWAS with *p*-values higher than random leading to false positive signals. Genomic control (GC) approach is extensively used to effectively control false positive signals. Genomic inflation factors ( λ ) can be computed as the median of the resulting chi-squared ( $\chi^{2}$ ) test statistics divided by the expected median of the chi-squared distribution. ^27^ Refer to equation 1 below

$$\lambda =\text{median}\left(\chi^{2}\right)/\text{qchisq}\left(0.5,1\right)$$ (1)

*Z* ( Beta/Se ), $\chi^{2}$ and *p*-value can be used to compute inflation factor, using $Z^{2}$ for Z , quantile of 1 - *p*-value at 1 degrees of freedom. The code below demonstrates how to compute inflation factors using the build-in R function qchisq that can be used to calculate value of quantile for a $\chi^{2}$ distribution.



# compute inflation factors
## use quantile function of chisq to p.value
data.gwas$p.value.qchisq <- qchisq(data.gwas$p.value, 1, lower.tail=FALSE)
## computed lambda
median(data, na.rm=TRUE)/qchisq(0.5, df)


**Global visualisation of results**. A common way to visualize GWAS results is the Manhattan plot. The Manhattan plot demonstrates the physical location of SNPs distributed by chromosome in the *x*-axis with the degree to which a SNP is associated with the target trait, i.e. *p*-value scaled by *log10* in the *y*-axis. A Manhattan plot can be done in *R* software using the qqman package, which includes functions for creating Manhattan plots and *q-q* plots from GWAS results. ^28^ The code below demonstrates how to create Manhattan plots, and *q-q* plots the qqman R package.



## do a qqplot and Manhattan plot using library qqman
library('qqman')
## used function qqplot from qqman
qqplot(data.gwas$p.value)
## used function manhattan from qqman
manhattan(data.gwas, chr="chr", bp="bp", p="p.value",snp="rsid")


**Local visualisation of results.** Local visualisation can be done using the method described by Pruim *et al*. ^29^ In this method, information of *LD*, genes, and previous results of GWAS can be added. Zoom version 2 offers a virtual analysis of local GWAS results. On the other hand, LocusTrack from the UCSC genome-browser adds more annotation compared to LocusZoom. ^30^ Furthermore, BigTop ^31^ is capable of providing three-dimensional visualisation of data using allele frequency as a third dimension. The code below demonstrates how to use LocusZoom to visualize SNPs considering LD information.



## use R to reformat your file to be used by locuszoom
R -e "library(data.table);
data.gwas<-fread('result.gwas');
data.gwas<-data.gwas[,c('chr','bp','bp','rsid','af','p.value')];
names(data.gwas)<-c('#CHROM', 'BEGIN', 'END', 'MARKER_ID', 'MAF', 'PVALUE');
write.table (data.gwas, file='gwas.epacts',row.names=F, col.names=T, sep='\t', quote=F)"
## use locus zoom to do a plot,
locuszoom/bin/locuszoom --epacts gwas.epacts \
--delim tab --refsnp rssnp \
--flank 10000 --pop EUR \
--build hg19 --source 1000G_Nov2014\
-p rs7412 --no-date \


### Gene-scoring approach

This approach considers the association between a trait and all SNPs within a predefined window around genes rather than each marker individually. In many cases, this approach is more powerful than traditional individual-SNP-based GWAS.

A gene score (GS) is a value given to a gene representing some measures related to a genetic trait. Thus, all the statistical summary values such as *p*-values and fold changes could be considered gene scoring values. For pGWAS analysis, GS is defined as the sum of all statistically significant alleles (i.e. the risk alleles) of the selected SNPs present in each individual under investigation. ^32^ Various algorithms have been developed to calculate GCs based on GWAS summary statistics. ^33- 35^ It is a common approach to encode and adjust SNPs values prior to calculating GC during analysis. ^32^ The process of SNP encoding aims to ensure that all SNPs are positively correlated with the outcome. ^32^ One more essential aspect to consider while calculating GS is the effect size. Variation in the effect size reflects reduction of predictive power of GS. ^32^ The GS method mostly used in pGWAS analysis is the gene-based *p*-value.

**Gene level *p*-value.** In many pGWAS pipelines, the first step in downstream GWAS analysis is to assign the SNPs to functional genomic features. The latter includes: coding genes, non-coding RNAs, 5’UTR, 3’UTR, proximal promoters, regulatory element, and enhancer elements.

Two common methods for assigning SNPs to their corresponding genes are GLOSSI ^36^ and VEGAS. ^34, 37^ GLOSSI is available as an R package, while VEGAS is available as an online tool as well as a stand-alone tool to be run on a local machine. Besides VEGAS and GLOSSI, many pGWAS tools provide procedures to calculate genes’ *p*-values. For instance, ancGWAS provides four different methods to calculate the genes *p*-values: Simes, Smallest, Fisher, and Gwbon. Similar to ancGWAS, MAGMA provides three methods to calculate genes’ *p*-values. However, one of the MAGMA methods is similar to the VEGAS method. The other two methods are by considering either the smallest SNP *p*-value, or the highest SNP *p*-values.

### Pathway-sub-network-based approaches

This approach considers the fact that complex biological phenomena addressed by GWAS, including the molecular basis of the rare disease, often arise due to gene interaction rather than single gene effect. Therefore, scientists analyse GWAS based on the biological network theory to understand the disease-causing genes and mechanisms involved in traits and complex diseases, such as in rare diseases. This approach is based on the results obtained from the gene-based association test and provides a higher level of complexity by considering biological networks and/or genes ontology. Analysing GWAS based on biological networks allows us to capture biological interactions between various molecules such as proteins, functional DNA motifs, coding and non-coding RNA, as well as disease mechanisms and to consider epigenetic changes, including methylation states or other modifications (phosphorylation, acetylation, etc). Also, this approach aims to map genes that are associated with significant SNPs into known pathways/Gene Ontology terms. The result of this approach provides information about the over-represented pathways in a given set of genes/SNPs.

### Fine-mapping and lead SNPs

GWAS will often identify a number of SNPs in *LD* with each other as being associated with the phenotype. The lead SNPs are those with the most significant *p*-value – they may be causal or not. The other SNPs in this region may only have an association because they are in *LD* with the causal SNP or they may be independently associated. This GWAS limitation may be observed when integrating information of variability in allele frequency, SNPs in *LD* block and imputation. ^38, 39^ For instance, when simulating GWAS results using OR value of 1.5 and allele frequency of 0.5 , only 21 % of the simulations demonstrated that the identified causal variants were not the most strongly associated variants. ^40^ On the other hand, changing the previous parameters *OR* value of 1.1 and allele frequency of 5 %, resulted in 2.4 % concordance between causal variants and lead SNPs. ^40^ Therefore, changing the previous parameters *OR* value of 1.1 and allele frequency of 5 %, resulted in 2.4 % concordance between causal variants and lead SNPs. ^40^

Therefore, fine-mapping methods are used to identify causal variants that are associated with the target trait and the number of putative causal variants from GWAS data. The fine-mapping approach integrates summary statistics from GWAS data, *LD* and functional annotations. There are two fine-mapping methods: (i) heuristic method that penalizes regression models, and (ii) Bayesian fine-mapping methods. ^38, 39^

**Lead SNPs.** Lead SNPs are defined as independent SNPs that have reached a minimum *p*-value threshold, i.e. they are independent of each other at the *LD* threshold. It is common to measure *LD* as $r^{2}$ where the square of the correlation coefficient between any two indicator variables is $r^{2}$ . ^41^ PLINK has implemented a function called’ *ld-clump*’ that clumps independent SNPs. ^15^ Moreover, FUMA tool ^42^ identifies lead SNPs by double clumping method. The first clumping is used for clumping SNPs with *p*-value < 0.05 at genome-wide significant *p*-value, i.e. *p*-value < 5e‐08 and independent at $r^{2}<$ 0.6 . This first clumping function reports significant independent SNPs. The second clumping is of significant independent SNPs at $r^{2}<$ 0.1 and reports lead SNPs. The code below demonstrates how to use the Plink tool to perform clumping to get lead SNPs.



## define lead snps using plink
# --clump-p1 Significance threshold for index SNPs
# --clump-p2 Secondary significance threshold for clumped SNPs
# --clump-r2 LD threshold for clumping
# --clump-kb Physical distance threshold for clumping
# --clump-snp-field your snp field must be same than in your assoc file
# --clump-field: p value header
plink --bfile plinkbase --clump result.gwas --clump-snp-field rsid\
--clump-field p.value --clump-p1 0.001 --clump-p2 0.1 \
--clump-r2 0.1 --clump-kb 250 --out assoc


**Heuristic fine-mapping approaches.** These are used to identify potential causal SNPs. ^38^ They are proposed to filter SNPs around lead SNPs considering the value of their pairwise correlation $r^{2}$ . They consider a hierarchical clustering technique to cluster all SNPs in a given region based on their pairwise $r^{2}$ . Another way is to use *LD* block by direct extraction of block and the selection of the same position on the block or by the visualisation representation of *LD* using LocusZoom ^29^ or Haploview. ^43^ Nonetheless, the described method is not a statistical approach to define putative causal variants, such as Bayesian methods or regression models. The code below demonstrates how to combine Plink tool and Haploview to perform heuristic fine-mapping.



### use haploview to plot your data
#### haploview has also an web interface
plink --keep list.ind -bfile $baseplink --recode tab --out fileres \
--chr chr --from-kb begin --to-kb end --maf 0.01 \
# execute Haploview in the previous range
java -jar Haploview.jar -n -minMAF $cut_maf -missingCutoff 0.01 \
-pedfile fileres.ped -map fileres.map -png -blockoutput


**Bayesian methods: framework.** The Bayesian method is commonly used to identify causal variants in a predefined SNPs window containing n number of SNPs. Knowing data ( D ) the Bayesian method aimed to maximize statistical model ( M ) using the following conditional probability

$$P\left(M|D\right)$$

Users first define an initial number of causal variant c (between 1,m SNPs). This number c is defined using genome-wide significant SNPs. To model Bayesian statistics, software will define a model M that contains c SNPs. This model often will restrain choice to the significant SNPs or suggest significant SNPs ( m ) in the windows. Considering the rule of combination $C_{m,c}=\frac{m!}{c!\left(n-c\right)!}$ , the probability model $P\left(D|M\right)$ is relatively easier to compute. The following Bayesian rules can be used

$$P\left(M|D\right)=\frac{P\left(D|M\right)}{P\left(M\right)P\left(D\right)}$$

Most of the tools are aimed to find a maximum probability to have the best combination of the causal variants. To model Bayesian statistics, many tools integrate various data such as *LD* and GWAS summary statistics.

**Bayesian methods: posterior inclusion probability.** This approach is used to compute post inclusion probability (PIP) at each SNP i . The PIP is computed by the sum of the posteriors over all models that include SNP i as a causal variant. ^38^

$${\mathrm{PIP}}_{i}=\sum_{i}\mathrm{mP}\left(M|D\right)$$ (2)

Using the rank of PIP is an excellent way to select putative causal variants. ^38^ The PIP approach should be used with caution to identify causal variants in the high *LD* regions. Therefore, it is recommended to estimate the posterior expected number of causal SNPs by summing the estimated PIPs for all SNPs in the region.

**Bayesian methods: credible sets.** This approach is used to define a set of variants that could have a good candidate. One way to estimate a credible set is to use PIP by ranking the values and doing the cumulative sum of PIP from the largest to the smallest. Then select all variants where the sum is less than the predefined α cutoff value. In general, researchers use 99% or 95% as a recommended value for α . Studies have shown that credible set and coverage probabilities are over-conservative in most fine-mapping situations as data sets are not randomly selected from among all causal variants. Therefore, an adjusted coverage is proposed to reduce such over-conservation in this approach. ^44^ The code below demonstrates how to combine Plink tool and FINEMAP to perform Bayesian-based fine-mapping analysis.



# extract a range of interest
plink -bfile $plk --keep-allele-order --chr chr
--from-kb begin --to-kb end --make-bed -out plink_range
#compute LD
plink --r2 square0 yes-really -bfile plink_range -out ld_range
# format ld
sed 's/\t/ /g' tmp.ld > $outld
## Finemaping
#create a config file
echo "z;ld;snp;config;cred;log;n_samples" \
> fileconfig.finemap
echo "$filez;$ld;${out}.snp;${out}.config;${out}.cred;${out}.log
;${params.n_pop}" >> $fileconfig
FINEMAP --cond --in-files fileconfig  --log \
--cond-pvalue 0.0000001 --n-causal-snps $ncausalsnp
### caviarbf
caviarbf -z ${filez} -r $ld -t 0 -a ${params.caviarbf_avalue} \
-c $ncausalsnp -o ${output} -n ${params.n_pop}
nb=`cat ${filez}|wc -l `
modelsearch -i $output -p 0 -o $output -m \$nb


**Bayesian methods: trans ethnic fine-mapping.** This is used to perform trans-ethnic fine-mapping studies using simulation results in similar fine-mapping resolution among the European and Asian ancestries. However, the inclusion of samples with African ancestry in meta-analysis leads to a significant improvement in fine-mapping resolution due to the lowest *LD* in the African ancestry population. The probability that the lead GWAS variants were also the causal variants increased using trans-ethnic GWAS data. ^40, 45^ It is a process that relies on disparate *LD* patterns in populations of diverse genetic ancestries to localize the causal variants. This approach has been successfully implemented to fine-map and leads to several common GWAS findings. ^46^

**Integrating annotation into fine-mapping.** Functional annotations have been shown to improve the discovery power and fine-mapping accuracy. Therefore, some tools such as CausalDB, ^47^ PAINTOR ^48^ and BIMBAM ^49^ have integrated expression quantitative trait locus (eQTL) information in fine-mapping approach.

**Bayesian methods: software.** Several Bayesian-based fine-mapping tools have been developed using summary statistics, *LD* and eQTL ( Table 1). Also, some pipelines have integrated different Bayesian-based software in order to compare their results (fine-mapping of h3agwas, fine-mapping in FinnGen, and FM pipeline).

**Table 1.** Examples of software used in fine-mapping based on Bayesian methods. More descriptives can be found in. ^38^

Software	Input	Output	Reference
FINEMAP	sumstat: beta, se, LD, n causal	PIP and Bayesian	Web Page ^50^
CaviarBF	sumstat: z value, LD, eQTL, fixed causal	PIP and Bayesian	Web Page ^47^
PAINTOR	sumstat: z value, LD, eQTL, fixed causal, multi LD	PIP and Bayesian	Web Page ^48^
CAVIAR - eCAVIAR	sumstat, LD, eQTL fixed causal	probability and confidence set	Web Page ^51, 52^

**Other fine-mapping approaches.** Other approaches, such as regression models, are used with all SNPs in the lead SNPs region to analyze SNPs jointly. The comparison of various approaches including elastic net, ridge, Lasso, MCP, and the normal-exponential shrinkage prior, have shown that penalized methods outperform single marker analysis. ^53^ Furthermore, a forward/stepwise regression can be used to test the independence of multi SNPs using the following algorithm:

• Order the list of SNPs by their *p*-values $\left[p_{0},p_{1},\ldots ,p_{n-1}\right]$

• Remove highest *p*-value
• Apply models with $\left[p_{1},\ldots ,p_{n-1}\right]$ with $p_{0}$ as a co-variate and check if any is significant
• Repeat process.

The *R* library SusieR has implemented a method of regression fine-mapping analyses.

**Other resources for fine-mapping analyses.** Several review articles have been published for fine-mapping analyses. Nevertheless, we recommend the following scientific articles as a good source for beginners: “From genome-wide associations to candidate causal variants by statistical fine-mapping”, ^38^ “A practical view of fine-mapping and gene prioritization in the post-genome-wide association era”, ^39^ and “Fine-mapping genetic associations”. ^54^

### Conditional association and imputation using summary statistics

The aim of performing conditional association and imputation using summary statistics is to evaluate the association between SNPs and biological trait by combining various GWAS summaries from different studies. This method requires a reference population to estimate *LD* information. The imputation method performs meta-analysis to infer the missing genotypes among the studies before evaluating the association between the SNPs and the biological trait. This method estimates the effects of many variants that are not directly genotyped.

### Polygenic predictions of disease risk

This method is used to predict disease risk using GWAS summaries. ^55^ Polygenic risk score (PRS) could be used to predict an individual’s likelihood to develop a specific trait or to estimate the level of predictive power that the trait is associated with a particular set of variants. ^56^ Although PRS methods are classified into Bayesian-based methods and non-Bayesian methods, there are more classifications of underlying PRS methods. ^57^ PRS is calculated by aggregating effects from a large set of causal SNPs. Several tools were developed to calculate PRS. For performing PRS studies, we highly recommend this recent review paper. ^58^ PRS is usually computed after the challenges associated with GWAS are carefully addressed. Here, we demonstrate key quality control ( *QC*) measures and include sample bash scripts. Table 2 summarizes the seven *QC* measures and contains the guidelines on specific thresholds. Thresholds can differ depending on the study’s unique features.

**Table 2.** Overview of seven quality control steps that should be conducted prior to polygenic risk score computation.

S/N	Step	Command	Function	Threshold and explanation
1	Missingness of SNPs and individuals	–geno –mind	SNPs with low genotype calls are removed.	Firstly, we suggest filtering SNPs and individuals on a relaxed threshold (0.2; >20% ), as this filters out SNPs and individuals with very high missingness.
2	Sex discrepancy	–check-sex	Checks for discrepancies between sex of the individuals recorded in the dataset and their sex based on X chromosome	If this discrepancy exists in many subjects, the data should be closely checked. Males should have an X chromosome homozygosity estimate >0.8 and females should have a value <0.2.
3	Minor allele frequency (MAF)	–maf	Includes only SNPs above the set MAF threshold.	Larger samples can use lower MAF thresholds, while smaller samples can use higher MAF thresholds. MAF thresholds of 0.01 and 0.05 are commonly used for high ( N = 100000) and moderate ( N = 10000) samples, respectively.
4	Hardy–Weinberg equilibrium (HWE)	–hwe	Excludes markers which deviate from Hardy–Weinberg equilibrium.	For binary traits we suggest excluding: HWE with *p*-value <1e‐01 in cases and <1e‐06 in controls. For quantitative traits, we recommend HWE *p*-value <1e-6.
5	Heterozygosity	x±3σ	Excludes individuals with high or low heterozygosity rates	We recommend excluding individuals that differ by ±3 SD from the heterozygozity rate mean in the samples.
6	Excludes individuals with high or low heterozygosity rates	–genome –min	Calculates identity by descent (IBD) of all sample pairs.	Use independent SNPs (pruning) for this analysis and limit it to autosomal chromosomes only.
7	Population stratification	–genome –cluster–cluster –mds-plot k	Produces a k-dimensional representation of any substructure in the data, based on IBS.	K is the number of dimensions, which needs to be defined (typically 10). This is an important step of the QC that consists of multiple proceedings.

**GWAS effect allele.** As datasets for PRS come from different GWAS experiments, it is critical to ensure consistency. Knowing which allele is considered the effect allele, it is vital to get an accurate PRS score. However, the effect allele is not labeled clearly in many datasets. ^59^ Different allele coding schemes exist, including Illumina’s TOP/BOTTOM coding concept, ALT/REF, effect/other, HapMap’s forward allele coding, Illumina’s A/B allele coding, Affymetrix’s A/B allele coding, REF (reference)/ALT (alternative), PLINK’s 1/2 allele coding, A1 (allele1)/A2 (allele2), A0 (allele 0)/A1 (allele1), effect allele/non-effect allele, effect allele/other allele, and many others. ^58, 59^ To avoid allele inconsistency, researchers should carefully read the documentation of GWAS datasets.

**SNPs level errors.** The *QC* assessment at the SNP level is crucial to avoid misleading PRS. SNPs level errors include (i) mismatching SNPs, i.e., inconsistent SNPs due to position difference in genomic position or nucleotide type, (ii) existence of duplicate SNP, (iii) ambiguous SNPs, i.e., researchers have no idea about SNP strand (ambiguous SNPs usually are C/G or A/T SNPs), and (iv) missing alleles.

**Chip heritability.** The chip heritability ( $h_{\mathrm{SNP}}^{2}$ )is also known as SNP-based heritability, which is defined as the portion of the phenotypic variation that the genotyped genetic marker can explain. ^60, 61^ Higher values of heritability indicate that the phenotype is explained best by the genotype, i.e. set of SNPs. Choi *et al.* ^58^ recommend $h_{\mathrm{SNP}}^{2}>0.05$ to perform PRS analysis. To estimate $h_{\mathrm{SNP}}^{2}$ , researchers should use *LD* score regression that could be used to distinguish polygenicity (SNPs effects) and confounding biases, including cryptic relatedness and population stratification. ^62^

The code below demonstrates how to use Plink to perform quality control checks and calculating PRS.



# $bfile: Target data set in Plink binary format.
# $QC: Base data set i.e., base GWAS summary statistic. This file contains P-value information
# $p1: P-value threshold for a SNP to be included as an index SNP. Choose value of 1 if you want to
##include all SNPs are include for clumping.
# $r2: Cutoff for r2 value i.e., SNPs having value higher than given r2 will be removed
# $kb: cutoff value window size in kilobase, i.e., SNPs within $kb of the index SNP are considered for clumping.
# $SNP: Name the column SNP that containing the SNP IDs
# $P: Name of the column that containing the P-value information
# $Output: Output file

################### Quality Control of Target Samples
plink\
  --bfile $bfile \
  --maf 0.05 \
  --mind 0.1 \
  --geno 0.1 \
  --hwe 1e-6 \
  --make-just-bim \
  --make-just-fam \
  --out $Output.qc

################### Clumping
plink \
  --bfile $bfile \
  --clump-p1 $p1 \
  --clump-r2 $r2 \
  --clump-kb $kb \
  --clump $QC \
  --clump-snp-field $SNP \
  --clump-field $P \
  --out $Output

############### calculating PRS
# $listpvalue: Threshold values
# $valid.snp: valid SNPs
# Here we are assuming that 3 column for SNP ID; 4 for effective allele information;
### the 12 for effect size estimate.
#### Moreover, this file has a header.

plink \
  --bfile $bfile \
  --score $QC 3 4 12 header \
  --q-score-range $listpvalue \
  --extract $valid.snp \
  --out $Output


### Meta-analysis

The meta-analysis approach can be used to evaluate the association between SNPs and biological traits by combining various GWAS summaries from different studies. The following paragraphs will provide the key concepts for performing a meta-analysis. To have concrete information about the meta-analysis approach, we recommend this review article. ^63^

**Heterogeneity of source.** Heterogeneity in data could be derived using GWAS summary statistics. The standard variables to estimate heterogeneity in data include odds ratios, standardized effect sizes, other metrics along with their uncertainty (e.g. variance or 95% confidence interval) and the accompanying *p*-values. However, there might be many other variables for each dataset that are important to deal with in order to estimate heterogeneity in data.

**Standard meta-analysis.** This is used to perform the meta-analysis approach which is to sum the *Z*-scores across all studies and weigh them appropriately using the sample sizes. See equation (3) below.

$$Z=\sum_{k=1}^{K}W_{k}Z_{k}$$ (3)

where $Z_{k}$ is Z -score from $K^{t}h$ study, and $w_{k}$ weight of studies relative to population size.

**Independence of the samples.** Conventional meta-analysis has an assumption that assumes that effect sizes are independent. Simulation studies demonstrate that failure to account for overlapping samples could greatly inflate type I error. ^64^ If accounting for the overlap is unavoidable, the overlap/covariance can be estimated using *Z*’s covariance between summary statistics. Some tools can account for overlapping samples, such as METAL software ^65^ and ASSET. ^66^

**Correcting for population structure with genomics control.** The presence of population structure in the GWAS study can impact an over-dispersion of the corresponding association test statistics. One approach to limit this problem is to correct statistics of each summary using genomic control. This correction factor is given as the inflation factor ( λ ) which is the test statistics’ median divided by its expectation under the null hypothesis. ^67^

***p*-values versus *Z* scores.** Meta-analysis methods based on *p*-values were widely used in different scientific fields until the 1980s . Then became unpopular and almost abandoned in biomedical sciences. Nowadays, the meta-analysis approach is performed using *Z*-score. There are two methods to estimate *Z*-score for GWAS data. The first method is demonstrated in equation (4).

Z=β/σ (4)

In the second method ( equation (5)), *Z*-score is estimated using *p*-value and the effect of allele.

$$Z_{j}=\Sigma^{-}1\left(P_{i}/2\right)*\mathrm{sign}\left(\Delta_{i}\right)$$ (5)

where $\text{sign}\left(\Delta_{i}\right)$ is a sign of relation.

**Random effects versus fixed effects.** In the presence of variability of allelic effect as in trans-ethnic studies, it is common to perform a random-effect meta-analysis to correct variability of β effect between different studies. For instance, GWAMA tool ^68^ computes a random-effects variance component using Cochran’s statistic ( *Q*-value) to balance weight used in meta-analysis. On the other hand, Metasoft ^69^ proposed two other different methods to take into account the heterogeneity, which are Random Effects model ^70^ and binary effects model with *m*-value, i.e. weight of each summary study in summary statistics. ^71^

**Heterogeneity test.** Heterogeneity at a locus can be reflected in the variability in population or environment. It can be relevant to gene-environment interaction and the reason behind the variability in GWAS approach of each data-set, i.e. covariable, model and approximation of GWAS between summary statistics. The heterogeneity is computed between two sets of summary statistics rather than one locus.

Cochran’s statistic provides a test of heterogeneity of allelic effects at SNPs j using equation (6) below.

$$Q_{j}=\sum_{i=1}^{N}W_{\mathrm{ij}}{\left(\beta_{j}-\beta_{i}j\right)}^{2}$$ (6)

where N denotes study number.

Alternatively, we use Q statistic to quantify the extent of heterogeneity in allelic effects across studies. ^72^ See equation (7) below

$$I^{2}=\left[Q_{j}-\left(N_{j}-1\right)\right]/Q_{j}$$ (7)

**Other meta-analysis approaches.** Traditionally, meta-analyses of GWAS have focused on combining results of multiple studies for similar traits. The Bayesian framework has been tested to estimate β on different phenotypes. ^73^ MetABF tool ^74^ has implemented a method to perform meta-analysis across genome-wide association studies of diverse phenotypes. It is important to note that a recent review on cancer suggests that it is possible to obtain “noteworthy” Bayesian results at higher *p*-values that are not considered statistically significant in GWAS. ^75^

**Study alignment and error trapping.** Meta-analysis aggregates various summary statistics. Therefore, any error to designate the effect allele and other allele or strand issue can cause an error in estimate β . Such errors might lead to misleading meta-analysis results as it increases Q of Cohran and heterogeneity between studies.

**Effect size.** By conducting a meta-analysis, researchers often neglect the sample size variation among different studies “true effect sizes are the same across studies”. However, in some cases, researchers introduce the correct effect size by considering the posterior probability for each study. Some software for estimating effect size are given in Table 3.

**Table 3.** List of software used in Meta-Analysis used for GWAS.

Software	specificity	remarks	link and publication
GWAMA	FE, RE, GC	short manual	Web Page ^50^
Meta-Soft	FE, RE, RE2, FE2 and BE, Q, $I^{2}$ GC	R script to plot effect of study	Web Page ^70^
MR-MEGA	FE, RE, Q, $I^{2}$	manual limited	Web Page ^76^
METAL	FE, RE, Q, $I^{2}$ , SOC, *p*-value, GC		Web Page ^65^
PLINK (1.9)	FE, RE	Few options described	Web Page ^15^

FE as fixed-effect, RE as random effect SOC as sample overlap correction, Q as Cochran’s statistic, I as Q statistic, and R as R-project library, GC as genomic control.

**Meta-analysis output.** Meta-analysis provides a new set of summary statistics. For each position that has not been discarded, new statistics will be calculated. These statistics include new values for β , σ and *p*-value. Users should be aware of the *LD* and the reference population used.

**More resources for meta-analysis.** We recommend h3bionet/h3agwas for meta-analysis pipeline. In addition, those interested in meta-analysis are directed to a review published by Zeggini *et al*. ^77^ and another review article by Evangelou and Ioannidis. ^63^ The code below demonstrates how to use the Metasoft tool to perform meta analysis.



# example with metasoft
## reformatting input files.
### File 1
R -e "library(data.table);data.gwas<-fread('$File 2');
data.gwas<-data.gwas[,c('CHR','SNP','BP','ALLELE1',
'ALLELE0','BETA','SE','P_BOLT_LMM')];
names(data.gwas)<-c('CHR', 'SNP','BP','A1',
'A2','BETA', 'SE','P');
data.gwas<-data.gwas[data.gwas[['P']]<1];
write.table(data.gwas, file='gwas_file2.qassoc',
row.names=F, col.names=T, sep='\t', quote=F)"
### File 2
R -e "library(data.table);data.gwas<-fread('$File 1');
data.gwas<-data.gwas[,c('CHR','SNP','BP','ALLELE1',
'ALLELE0','BETA','SE','P_BOLT_LMM')];
names(data.gwas)<-c('CHR', 'SNP','BP','A1',
'A2','BETA', 'SE','P');
data.gwas<-data.gwas[data.gwas[['P']]<1];
write.table(data.gwas, file='gwas_file1.qassoc',
row.names=F, col.names=T, sep='\t', quote=F)"
## metasoft
### Metasoft with binary effect
java -jar Metasof/Metasoft.jar \
-input file_merge_all.meta\
-output meta.meta \
-pvalue_table Metasof/HanEskinPvalueTable.txt\
-binary_effects\


### Colocalization analysis

**Colocalization.** Colocalization is an approach used to integrate annotations with GWAS results. The annotations resources include gene expression (eQTLs), protein expression (pQTLs), exon splicing (sQTLs), DNA methylation (mQTLs), and chromatin acetylation and chromatin accessibility (caQTLs). ^78^

**Statistics for colocalization studies.** Several parametric and non-parametric statistics can be done for colocalization studies. ^79^

**Resources for colocalization studies.** We recommend the following scientific resources for those who are beginners in this field:

• From GWAS to function: using functional genomics to identify the mechanisms underlying complex diseases. ^78^
• Colocalization analyses of genomic elements: approaches, recommendations and challenges. ^79^
• Bayesian test for colocalisation between pairs of genetic association studies using summary statistics. ^80^
• LocusFocus: web-based colocalization for the annotation and functional follow-up of GWAS colocalization of GWAS and eQTL signals detects target genes. ^81^
• A powerful and versatile colocalization test. ^82^

**Using summary statistics from multiple phenotypes and traits.** The methods for GWAS are mostly focused on single variant analysis with a single phenotype or trait. Increasing evidence shows that pleiotropy, one gene’s effect on multiple phenotypes, plays a pivotal role in many complex traits. Therefore, associating different GWAS results for multiple phenotypes can provide an extensive power by aggregating multiple weak signals. ^83^ Different approaches have been developed to integrate dependent *p*-values to assess the association between a gene and multiple correlated phenotypes. ^83^ Several tools exist to perform multi traits analysis, including Multi-Trait Analysis of GWAS (MTAG) ^84^ and CPASSOC package. ^85^

### Mendelian randomisation

Mendelian randomisation (MR) is a statistical approach that can be defined as “the use of genetic variants as instrumental variables to investigate the effects of modifiable risk factors for disease”. ^86^ For instance, one trait (phenotype or disease) might be affected by confounding or reverse causation rather than a conventional observational variable. Therefore, MR aims to provide a statistical frame to verify the causality between locus and phenotype and exclude pleiotropy. Such methods will provide a reliable explanation of the results.

**Assumption of MR.** There are three main assumptions for MR. ^87- 90^ These are (i) the genetic variant that is associated with the exposure (significant association), (ii) the genetic variant that is independent of the outcome given to the exposure and all confounders (measured and unmeasured) of the exposure-outcome association, (iii) the genetic variant that is independent of factors (measured and unmeasured) that confound the exposure-outcome relationship.

**Statistical methods for MR.** MR general strategy is to compare beta values or *p*-value of the same position of two or more different GWAS using related/confounding phenotype. Several methodologies have been developed for MR analysis, an example is the ratio of coefficients estimator, which can be modeled using equation (8):

$$\beta_{\text{ratio}}=\frac{\beta_{e}}{\beta_{o}}$$ (8)

where $\beta_{e}$ represents the change in exposure per variant allele, and $\beta_{o}$ represents the change in outcome per variant allele. Another model is the two-stage least squares. It employs a two-stage regression approach with two regression models where the first stage regression’s output is used as the input of the second stage regression. More methods for MR exist, including control function estimator, limited information maximum likelihood method, verse variance weighted method, and MR-Egger method. ^91^

**MR software.** Several tools have been developed to compute MR, e.g GSMR (Generalised Summary-data-based Mendelian Randomisation) from GCTA, TwoSampleMR (version 0.4.20), MR-PRESSO, and MR-LDP which is integrated LD information. We recommend the following tutorial: https://bioconductor.org/packages/release/bioc/vignettes/GMRP/inst/doc/GMRP.pdf. ^92^

The code below demonstrates how to use gcta64 tool to perform Mendelian randomisation analysis.



#reformat file 1
R -e "library(data.table);
data.gwas<-fread('$File');
data.gwas<-data.gwas[,c('SNP','ALLELE1','ALLELE0','A1FREQ','BETA','SE','P_BOLT_LMM')];
names(data.gwas)<-c('SNP','A1', 'A2','freq','b', 'se','p');
data.gwas[['N']]<-10000;
data.gwas<-data.gwas [data.gwas[['p']]<1];
SNPtmp<-table(data.gwas[['SNP']]);
UniqSNP<-names(SNPtmp)[SNPtmp==1];
write.table(data.gwas[data.gwas[['SNP']] %in% UniqSNP],file='FilePheno1',row.names=F,
col.names=T, sep='\t', quote=F)"

#reformat file 2
R -e "library(data.table);
data.gwas<-fread('$File');
data.gwas<-data.gwas[,c('SNP','ALLELE1','ALLELE0','A1FREQ','BETA','SE','P_BOLT_LMM')];
names(data.gwas)<-c('SNP','A1', 'A2','freq','b', 'se','p');
data.gwas[['N']]<-10000;
data.gwas<-data.gwas[data.gwas[['p']]<1];
SNPtmp<-table(data.gwas[['SNP']]);
UniqSNP<-names(SNPtmp)[SNPtmp==1];
write.table(data.gwas [data.gwas[['SNP']] \%in% UniqSNP],
file='FilePheno2',row.names=F, col.names=T,
sep='\t', quote=F)
# create
echo "P1 FilePheno1" > exposure
echo "Exp FilePheno2" > outcome

## gcta need a plink file or bgen file
## GSMR analyses,
###forward-GSMR analysis (coded as 0),
###reverse-GSMR analysis (coded as 1)
###and bi-GSMR analysis (both forward- and reverse-GSMR analyses, coded as 2).
gcta64 --bfile plkfile --gsmr-file exposure outcome \
--gsmr-direction 0 --out test_gsmr_result


**MR and gene expression.** Recently, some methods have integrated MR into GWAS and eQTL to test if the effect of gene expression is zero on the trait. ^93- 95^ These methods provide a promising way to combine GWAS summary statistics and expression data.

## Our recommended pGWAS pipeline

**Figure 1.**
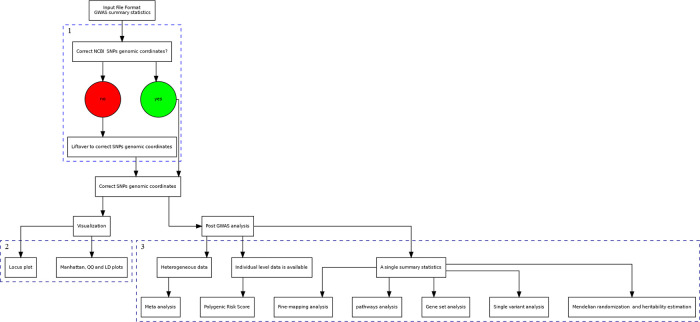
A general pGWAS pipeline consi sts of three main steps: Step 1 aims to perform data preprocessing, Step 2 for data visualization, and Step 3 for downstream pGWAS analysis.

Our proposed pGWAS pipeline consists of three main steps: preprocessing, visualization, and the downstream pGWAS analysis (refer to Figure 1).

Step *1*, the preprocessing step, aims to control checks and ensure a correct input file format for the downstream pGWAS analysis. The main purpose of this step is to ensure that SNPs’ positions in the GWAS summary file accurately match the genomic coordinates in the downstream reference panel if any is available. The UCSC LiftOver tool ( http://genome.ucsc.edu/cgi-bin/hgLiftOver) is widely used to correct genomic position mismatches between the GWAS summary file and the reference panel. However, other options exist, including: Bioconductor rtracklayer package, ^96^ Assembly Converter, ^97^ NCBI Remap, ^98^ and the CrossMap tool. ^99^

Step *2*, the visualization step, aims to visualize the raw input GWAS summary data pictorially, primarily through two scatter plots: Manhattan plot and quantile-quantile (Q-Q) plot. The Manhattan plot is widely used in genomics to visualize the results of GWAS studies. In the Manhattan plot, the X-axis represents the positions on chromosomes, while the Y-axis reflects genomic association strength with a given trait. The *Q-Q* plot is used to check the normality of data -mainly the normality of *p*-values distribution. Step *2* can be completed using the *qqman* R package. ^100^

Step *3*, of downstream pGWAS analysis, can be divided into three approaches based on their underlining data heterogeneity. First, if there is homogenous data, researchers can perform a single variant pGWAS analysis such as $\chi^{2}$ to test the association between a particular variant and trait. Furthermore, researchers can perform gene set analysis or network/pathway analysis to understand the biological function underlying a list of statistically significant variants. For instance, the MAGMA tool can be used to conduct gene set-based pGWAS research, ^101^ while pathway analysis could be done using the PASCAL (Pathway scoring algorithm) tool. ^102^ Researchers can also undertake fine-mapping analysis and MR for homogenous GWAS summary files. Second, researchers can perform PRS analysis and genetic risk score if individual-level data is available. Third, if there is heterogeneous data from numerous independent studies, a meta-analysis can be performed.

## Final remarks

This articles demonstrates various pGWAS methods. The advancement in these pGWAS techniques solves a significant problem in our efforts to understand the vast amount of data generated and explore fundamental biology. However, several issues should be taken into consideration when performing pGWAS analysis across trans-ethnic GWAS studies. Some of these issues include: (i) heritability of the trait, (ii) GWAS sample size, (iii) polygenicity of the traits, (iv) genetic architecture of the trait, and (v) genotype-environments interactions. Furthermore, we expect that in the future many pGWAS methods will be developed to address these limitations either for a particular ethnic group or for multi-ethnic groups.

## Data availability

No data is associated with this article.

## References

[1] Jia P Zheng S Long J : dmGWAS: dense module searching for genome-wide association studies in protein–protein interaction networks. *Bioinformatics.* jan 2011;27(1):95–102. 10.1093/bioinformatics/btq615 21045073 PMC3008643

[2] Edwards AO Ritter R Abel KJ : Complement factor H polymorphism and age-related macular degeneration. *Science.* 2005;308:421–424. 10.1126/science.1110189 15761121

[3] Tam V Patel N Turcotte M : Benefits and limitations of genome-wide association studies. 2019. 31068683 10.1038/s41576-019-0127-1

[4] Ball RD : Experimental designs for robust detection of effects in genome-wide case-control studies. *Genetics.* 2011;189:1497–1514. 10.1534/genetics.111.131698 21926296 PMC3241427

[5] Cantor RM Lange K Sinsheimer JS : Prioritizing GWAS Results: A Review of Statistical Methods and Recommendations for Their Application. 2010. 20074509 10.1016/j.ajhg.2009.11.017 PMC2801749

[6] Zhang Q Long Q Ott J : AprioriGWAS, a New Pattern Mining Strategy for Detecting Genetic Variants Associated with Disease through Interaction Effects. *PLoS Comput. Biol.* 2014;10:e1003627. 10.1371/journal.pcbi.1003627 24901472 PMC4046917

[7] Peng G Luo L Siu H : Gene and pathway-based second-wave analysis of genome-wide association studies. *Eur. J. Hum. Genet.* 2010;18:111–117. 10.1038/ejhg.2009.115 19584899 PMC2987176

[8] Arnau-Soler A Macdonald-Dunlop E Adams MJ : Genome-wide by environment interaction studies of depressive symptoms and psychosocial stress in UK biobank and generation scotland. *Transl. Psych.* February 2019;9(1):14. 10.1038/s41398-018-0360-y 30718454 PMC6361928

[9] Wang K Li M Hakonarson H : ANNOVAR: Functional annotation of genetic variants from high-throughput sequencing data. *Nucleic Acids Res.* 2010;38:e164. 10.1093/nar/gkq603 20601685 PMC2938201

[10] Shahbaba B Shachaf CM Zhaoxia Y : A pathway analysis method for genome-wide association studies. *Stat. Med.* 2012;31:988–1000. 10.1002/sim.4477 22302470

[11] Chimusa ER Daya M Möller M Ramesar R : Determining Ancestry Proportions in Complex Admixture Scenarios in South Africa Using a Novel Proxy Ancestry Selection Method. *PLoS One.* 2013;8:e73971. 10.1371/journal.pone.0073971 24066090 PMC3774743

[12] Pasaniuc B Price AL : Dissecting the genetics of complex traits using summary association statistics. *Nat. Rev. Genet.* feb 2017;18(2):117–127. 10.1038/nrg.2016.142 27840428 PMC5449190

[13] Turner S Armstrong LL Bradford Y : Quality control procedures for genome-wide association studies. *Curr. Protoc. Hum. Genet.* 2011;68. 10.1002/0471142905.hg0119s68 PMC3066182 21234875

[14] Wang MH Cordell HJ Van Steen K : Statistical methods for genome-wide association studies. *Semin. Cancer Biol.* apr 2019;55:53–60. 10.1016/j.semcancer.2018.04.008 29727703

[15] Purcell S Neale B Todd-Brown K : PLINK: A Tool Set for Whole-Genome Association and PopulationBased Linkage Analyses. *Am. J. Hum. Genet.* 2007;81:559–575. 10.1086/519795 17701901 PMC1950838

[16] Grady BJ Torstenson E Dudek SM : Finding unique filter sets in plato: a precursor to efficient interaction analysis in gwas data. *Pac. Symp. Biocomput.* 2010. PMC2903053 19908384

[17] Price AL Patterson NJ Plenge RM : Principal components analysis corrects for stratification in genome-wide association studies. *Nat. Genet.* 2006;38:904–909. 10.1038/ng1847 16862161

[18] Raj A Stephens M Pritchard JK : FastSTRUCTURE: Variational inference of population structure in large SNP data sets. *Genetics.* 2014;197:573–589. 10.1534/genetics.114.164350 24700103 PMC4063916

[19] R Core Team: *R: A Language and Environment for Statistical Computing.* Vienna, Austria: R Foundation for Statistical Computing;2018.

[20] Benjamini Y Hochberg Y : Controlling the false discovery rate: A practical and powerful approach to multiple testing. *J. Royal Statistical Society. Series B (Methodological).* 1995;57(1):289–300. 10.1111/j.2517-6161.1995.tb02031.x

[21] Pe’er I Yelensky R Altshuler D : Estimation of the multiple testing burden for genomewide association studies of nearly all common variants. *Genet. Epidemiol.* May 2008;32(4):381–385. 10.1002/gepi.20303 18348202

[22] Fadista J Manning AK Florez JC : The (in) famous GWAS P-value threshold revisited and updated for low-frequency variants. *Euro. J. Hum. Gene. EJHG.* 2016;24(8):1202–1205. 10.1038/ejhg.2015.269 26733288 PMC4970684

[23] Panagiotou OA Ioannidis JPA : for the Genome-Wide Significance Project: What should the genome-wide significance threshold be? Empirical replication of borderline genetic associations. *Int. J. Epidemiol.* 12 2011;41(1):273–286. 10.1093/ije/dyr178 22253303

[24] Kanai M Tanaka T Okada Y : Empirical estimation of genome-wide significance thresholds based on the 1000 Genomes Project data set. *J. Hum. Genet.* October 2016;61(10):861–866. 10.1038/jhg.2016.72 27305981 PMC5090169

[25] Gurdasani D Carstensen T Fatumo S : Uganda genome resource enables insights into population history and genomic discovery in africa. *Cell.* October 2019;179(4):984—1002.e36. 10.1016/j.cell.2019.10.004 31675503 PMC7202134

[26] Duggal P Gillanders EM Holmes TN : Establishing an adjusted p-value threshold to control the family-wide type 1 error in genome wide association studies. *BMC Genomics.* October 2008;9:516. 10.1186/1471-2164-9-516 18976480 PMC2621212

[27] Yang J Weedon MN Purcell S : Genomic inflation factors under polygenic inheritance. *Eur. J. Hum. Genet.* July 2011;19(7):807–812. 10.1038/ejhg.2011.39 21407268 PMC3137506

[28] Grace C Farrall M Watkins H : Manhattan++: displaying genome-wide association summary statistics with multiple annotation layers. *BMC Bioinform.* November 2019;20(1):610. 10.1186/s12859-019-3201-y 31775616 PMC6882345

[29] Pruim RJ Welch RP Sanna S : LocusZoom: regional visualization of genome-wide association scan results. *Bioinformatics (Oxford, England).* September 2010;26(18):2336–2337. 10.1093/bioinformatics/btq419 20634204 PMC2935401

[30] Cuellar-Partida G Renteria ME MacGregor S : LocusTrack: Integrated visualization of GWAS results and genomic annotation. *Source Code Biol. Med.* February 2015;10(1):1. 10.1186/s13029-015-0032-8 25750659 PMC4351846

[31] Westreich ST Nattestad M Meyer C : BigTop: a three-dimensional virtual reality tool for GWAS visualization. *BMC Bioinform.* January 2020;21(1):39. 10.1186/s12859-020-3373-5 32005132 PMC6995189

[32] Shabana SUS Hasnain S : Use of a gene score of multiple low-modest effect size variants can predict the risk of obesity better than the individual SNPs. *Lipids Health Dis.* July 2018;17(1):155. 10.1186/s12944-018-0806-5 30021629 PMC6052513

[33] Lamparter D Marbach D Rueedi R : Fast and rigorous computation of gene and pathway scores from SNP-based summary statistics. *PLoS Comput. Biol.* January 2016;12(1):e1004714. 10.1371/journal.pcbi.1004714 26808494 PMC4726509

[34] Liu JZ McRae AF Nyholt DR : A versatile gene-based test for genome-wide association studies. *Am. J. Hum. Genet.* 2010;87:139–145. 10.1016/j.ajhg.2010.06.009 20598278 PMC2896770

[35] Li M-X Gui H-S Kwan JSH : GATES: A rapid and powerful gene-based association test using extended simes procedure. *Am. J. Hum. Genet.* March 2011;88(3):283–293. 10.1016/j.ajhg.2011.01.019 21397060 PMC3059433

[36] Chai HS Sicotte H Bailey KR : GLOSSI: A method to assess the association of genetic loci-sets with complex diseases. *BMC Bioinform.* 2009;10. 10.1186/1471-2105-10-102 19344520 PMC2678095

[37] Mishra A Macgregor S : VEGAS2: Software for more flexible gene-based testing. *Twin Res. Hum. Genet.* 2015;18:86–91. 10.1017/thg.2014.79 25518859

[38] Schaid DJ Chen W Larson NB : From genome-wide associations to candidate causal variants by statistical fine-mapping. Nature reviews. *Genetics.* August 2018;19(8):491–504. 10.1038/s41576-018-0016-z 29844615 PMC6050137

[39] Broekema RV Bakker OB Jonkers IH : A practical view of fine-mapping and gene prioritization in the post-genomewide association era. *Open Biol.* 2020;10(1):190221. 10.1098/rsob.190221 31937202 PMC7014684

[40] Bunt M van de Adrian C : IGAS Consortium: Evaluating the Performance of Fine-Mapping Strategies at Common Variant GWAS Loci. *PLoS Genet.* 2015;11(9):e1005535. 10.1371/journal.pgen.1005535 26406328 PMC4583479

[41] VanLiere JM Rosenberg NA : Mathematical properties of the measure of linkage disequilibrium. *Theor. Popul. Biol.* August 2008;74(1):130–137. 10.1016/j.tpb.2008.05.006 18572214 PMC2580747

[42] Watanabe K Taskesen E Bochoven A van : Functional mapping and annotation of genetic associations with FUMA. *Nat. Commun.* November 2017;8(1):1826. 10.1038/s41467-017-01261-5 29184056 PMC5705698

[43] Barrett JC Fry B Maller J : Haploview: analysis and visualization of LD and haplotype maps. *Bioinformatics (Oxford, England).* January 2005;21(2):263–265. 10.1093/bioinformatics/bth457 15297300

[44] Hutchinson A Watson H Wallace C : Improving the coverage of credible sets in Bayesian genetic fine-mapping. *PLoS Comput. Biol.* April 2020;16(4).e1007829. 10.1371/journal.pcbi.1007829 32282791 PMC7179948

[45] Asimit JL Hatzikotoulas K McCarthy M : Trans-ethnic study design approaches for fine-mapping. *Eur. J. Hum. Genet.* September 2016;24(9):1330–1336. 10.1038/ejhg.2016.1 26839038 PMC4856879

[46] Xu W Teo Y-Y : Trans-Ethnic Fine-Mapping of Rare Causal Variants. Zeggini E Morris A , editors. *Assessing Rare Variation in Complex Traits: Design and Analysis of Genetic Studies.* New York, NY: Springer;2015; pages253–261. 10.1007/978-1-4939-2824-8_18

[47] Chen W Larrabee BR Ovsyannikova IG : Fine Mapping Causal Variants with an Approximate Bayesian Method Using Marginal Test Statistics. *Genetics.* July 2015;200(3):719–736. 10.1534/genetics.115.176107 25948564 PMC4512539

[48] Gong Y Greenbaum J Deng H-W : A statistical approach to fine-mapping for the identification of potential causal variants related to human intelligence. *J. Hum. Genet.* August 2019;64(8):781–787. 10.1038/s10038-019-0623-3 31165785 PMC6712985

[49] Servin B Stephens M : Imputation-based analysis of association studies: candidate regions and quantitative traits. *PLoS Genet.* July 2007;3(7):e114. 10.1371/journal.pgen.0030114 17676998 PMC1934390

[50] Mägi R Morris AP : GWAMA: software for genome-wide association meta-analysis. *BMC Bioinform.* May 2010;11:288. 10.1186/1471-2105-11-288 20509871 PMC2893603

[51] Hormozdiari F Bunt M van de Segrè AV : Colocalization of GWAS and eQTL Signals Detects Target Genes. *Am. J. Hum. Genet.* December 2016;99(6):1245–1260. 10.1016/j.ajhg.2016.10.003 27866706 PMC5142122

[52] Hormozdiari F Kostem E Kang EY : Identifying Causal Variants at Loci with Multiple Signals of Association. *Genetics.* October 2014;198(2):497–508. 10.1534/genetics.114.167908 25104515 PMC4196608

[53] Ayers KL Cordell HJ : SNP Selection in Genome-Wide and Candidate Gene Studies via Penalized Logistic Regression. *Genet. Epidemiol.* December 2010;34(8):879–891. 10.1002/gepi.20543 21104890 PMC3410531

[54] Hutchinson A Asimit J Wallace C : Fine-mapping genetic associations. *Hum. Mol. Genet.* 08 2020;29(R1):R81–R88. 10.1093/hmg/ddaa148 32744321 PMC7733401

[55] Pain O Glanville KP Hagenaars S : Evaluation of Polygenic Prediction Methodology within a Reference-Standardized Framework. *bioRxiv.* July 2020; page2020.07.28.224782. 10.1371/journal.pgen.1009021 PMC8121285 33945532

[56] Igo RP Kinzy TG Cooke Bailey JN : Genetic risk scores. *Curr. Protoc. Hum. Genet.* November 2019;104(1):e95. 10.1002/cphg.95 31765077 PMC6941594

[57] Adam Y Sadeeq S Kumuthini J : Polygenic risk score in africa population: Progress and challenges. 2021. 37273966 10.12688/f1000research.76218.2 PMC10233318

[58] Choi SW Mak TS-H O’Reilly PF : Tutorial: a guide to performing polygenic risk score analyses. *Nat. Protoc.* September 2020;15(9):2759–2772. 10.1038/s41596-020-0353-1 32709988 PMC7612115

[59] Wootton RE Sallis HM : Let’s call it the effect allele: a suggestion for GWAS naming conventions. *Int. J. Epidemiol.* September 2020;49(5):1734–1735. 10.1093/ije/dyaa149 32879951

[60] Yang J Lee SH Goddard ME : GCTA: A tool for genome-wide complex trait analysis. *Am. J. Hum. Genet.* January 2011;88(1):76–82. 10.1016/j.ajhg.2010.11.011 21167468 PMC3014363

[61] Sun J Kranzler HR Bi J : Refining multivariate disease phenotypes for high chip heritability. *BMC Med. Genet.* September 2015;8(S3). 10.1186/1755-8794-8-S3-S3 PMC4582350 26399736

[62] Bulik-Sullivan BK Loh P-R Finucane HK : LD score regression distinguishes confounding from polygenicity in genome-wide association studies. *Nat. Genet.* February 2015;47(3):291–295. 10.1038/ng.3211 25642630 PMC4495769

[63] Evangelou E Ioannidis JPA : Meta-analysis methods for genome-wide association studies and beyond. *Nat. Rev. Genet.* May 2013;14(6):379–389. 10.1038/nrg3472 23657481

[64] Lin D-Y Sullivan PF : Meta-Analysis of Genome-wide Association Studies with Overlapping Subjects. *Am. J. Hum. Genet.* December 2009;85(6):862–872. 10.1016/j.ajhg.2009.11.001 20004761 PMC2790578

[65] Willer CJ Li Y Abecasis GR : METAL: fast and efficient meta-analysis of genomewide association scans. *Bioinformatics (Oxford, England).* September 2010;26(17):2190–2191. 10.1093/bioinformatics/btq340 20616382 PMC2922887

[66] Bhattacharjee S Rajaraman P Jacobs KB : A subset-based approach improves power and interpretation for the combined analysis of genetic association studies of heterogeneous traits. *Am. J. Hum. Genet.* May 2012;90(5):821–835. 10.1016/j.ajhg.2012.03.015 22560090 PMC3376551

[67] Devlin B Roeder K : Genomic control for association studies. *Biometrics.* December 1999;55(4):997–1004. 10.1111/j.0006-341X.1999.00997.x 11315092

[68] Mägi R Morris AP : GWAMA: software for genome-wide association meta-analysis. *BMC Bioinform.* May 2010;11(1). 10.1186/1471-2105-11-288 PMC2893603 20509871

[69] Han B Eskin E : Random-effects model aimed at discovering associations in meta-analysis of genome-wide association studies. *Am. J. Hum. Genet.* May 2011;88(5):586–598. 10.1016/j.ajhg.2011.04.014 21565292 PMC3146723

[70] Han B Eskin E : Random-Effects Model Aimed at Discovering Associations in Meta-Analysis of Genome-wide Association Studies. *Am. J. Hum. Genet.* May 2011;88(5):586–598. 10.1016/j.ajhg.2011.04.014 21565292 PMC3146723

[71] Han B Eskin E : Interpreting Meta-Analyses of Genome-Wide Association Studies. *PLoS Genet.* March 2012;8(3):e1002555. 10.1371/journal.pgen.1002555 22396665 PMC3291559

[72] Huedo-Medina TB Sánchez-Meca J Marín-Martínez F : Assessing heterogeneity in metaanalysis: Q statistic or I2 index?. *Psychol. Meth.* June 2006;11(2):193–206. 10.1037/1082-989X.11.2.193 16784338

[73] Trochet H Pirinen M Band G : Bayesian meta-analysis across genome-wide association studies of diverse phenotypes. *Genet. Epidemiol.* 2019;43(5):532–547. 10.1002/gepi.22202 30920090

[74] Trochet H Pirinen M Band G : Bayesian meta-analysis across genome-wide association studies of diverse phenotypes. *Genet. Epidemiol.* March 2019;43(5):532–547. 10.1002/gepi.22202 30920090

[75] Park JH Geum DI Eisenhut M : Bayesian statistical methods in genetic association studies: Empirical examination of statistically non-significant Genome Wide Association Study (GWAS) meta-analyses in cancers: A systematic review. *Gene.* February 2019;685:170–178. 10.1016/j.gene.2018.10.057 30416053

[76] Mägi R Horikoshi M Sofer T : Trans-ethnic meta-regression of genome-wide association studies accounting for ancestry increases power for discovery and improves fine-mapping resolution. *Hum. Mol. Genet.* September 2017;26(18):3639–3650. 10.1093/hmg/ddx280 28911207 PMC5755684

[77] Zeggini E Ioannidis JPA : Meta-analysis in genome-wide association studies. *Pharmacogenomics.* February 2009;10(2):191–201. 10.2217/14622416.10.2.191 19207020 PMC2695132

[78] Cano-Gamez E Trynka G : From GWAS to Function: Using Functional Genomics to Identify the Mechanisms Underlying Complex Diseases. *Front. Genet.* 2020;11. 10.3389/fgene.2020.00424 PMC7237642 32477401

[79] Kanduri C Bock C Gundersen S : Colocalization analyses of genomic elements: approaches, recommendations and challenges. *Bioinformatics.* May 2019;35(9):1615–1624. 10.1093/bioinformatics/bty835 30307532 PMC6499241

[80] Giambartolomei C Vukcevic D Schadt EE : Bayesian test for colocalisation between pairs of genetic association studies using summary statistics. *PLoS Genet.* May 2014;10(5):e1004383. 10.1371/journal.pgen.1004383 24830394 PMC4022491

[81] Panjwani N Wang F Mastromatteo S : LocusFocus: Web-based colocalization for the annotation and functional follow-up of GWAS. *PLoS Comput. Biol.* October 2020;16(10):e1008336. 10.1371/journal.pcbi.1008336 33090994 PMC7608978

[82] Deng Y Pan W : A powerful and versatile colocalization test. *PLoS Comput. Biol.* April 2020;16(4):e1007778. 10.1371/journal.pcbi.1007778 32275709 PMC7176287

[83] Deng Y He T Fang R : Genome-Wide Gene-Based Multi-Trait Analysis. *Front. Genet.* May 2020;11. 10.3389/fgene.2020.00437 PMC7248273 32508874

[84] Turley P Walters RK Maghzian O : Multi-trait analysis of genome-wide association summary statistics using MTAG. *Nat. Genet.* February 2018;50(2):229–237. 10.1038/s41588-017-0009-4 29292387 PMC5805593

[85] Zhu X Feng T Tayo BO : Meta-analysis of Correlated Traits via Summary Statistics from GWASs with an Application in Hypertension. *Am. J. Hum. Genet.* January 2015;96(1):21–36. 10.1016/j.ajhg.2014.11.011 25500260 PMC4289691

[86] Davies NM Holmes MV Smith GD : Reading Mendelian randomisation studies: a guide, glossary, and checklist for clinicians. *BMJ.* July 2018;362:k601. 10.1136/bmj.k601 30002074 PMC6041728

[87] Teumer A : Common Methods for Performing Mendelian Randomization. *Front. Cardio. Med.* May 2018;5. 10.3389/fcvm.2018.00051 PMC5985452 29892602

[88] Glymour MM Tchetgen Tchetgen EJ Robins JM : Credible Mendelian Randomization Studies: Approaches for Evaluating the Instrumental Variable Assumptions. *Am. J. Epidemiol.* February 2012;175(4):332–339. 10.1093/aje/kwr323 22247045 PMC3366596

[89] Didelez V Meng S Sheehan NA : Assumptions of IV Methods for Observational Epidemiology. *Stat. Sci.* February 2010;25(1):22–40. 10.1214/09-STS316

[90] Bowden J Smith GD Burgess S : Mendelian randomization with invalid instruments: effect estimation and bias detection through Egger regression. *Int. J. Epidemiol.* April 2015;44(2):512–525. 10.1093/ije/dyv080 26050253 PMC4469799

[91] Grover S Fabiola Del Greco M Stein CM : Mendelian Randomization. Elston RC , editor. *Statistical Human Genetics: Methods and Protocols, Methods in Molecular Biology.* New York, NY: Springer;2017; pages581–628. 10.1007/978-1-4939-7274-6_29 28980266

[92] Cheng Q Yang Y Shi X : MR-LDP: a two-sample Mendelian randomization for GWAS summary statistics accounting for linkage disequilibrium and horizontal pleiotropy. *NAR Geno. Bioinform.* June 2020;2(lqaa028). 10.1093/nargab/lqaa028 PMC7671398 33575584

[93] Porcu E Rüeger S Lepik K : Mendelian randomization integrating GWAS and eQTL data reveals genetic determinants of complex and clinical traits. *Nat. Commun.* July 2019;10(1):3300. 10.1038/s41467-019-10936-0 31341166 PMC6656778

[94] Richardson TG Hemani G Gaunt TR : A transcriptome-wide Mendelian randomization study to uncover tissue-dependent regulatory mechanisms across the human phenome. *Nat. Commun.* January 2020;11(1):185. 10.1038/s41467-019-13921-9 31924771 PMC6954187

[95] Gleason KJ Yang F Chen LS : A robust two-sample Mendelian Randomization method integrating GWAS with multi-tissue eQTL summary statistics. *bioRxiv.* June 2020; page2020.06.04.135541. 10.1002/gepi.22380 PMC9027205 33834509

[96] Lawrence M Gentleman R Carey V : rtracklayer: an r package for interfacing with genome browsers. *Bioinformatics.* May 2009;25(14):1841–1842. 10.1093/bioinformatics/btp328 19468054 PMC2705236

[97] Howe KL Achuthan P Allen J : Ensembl 2021. *Nucleic Acids Res.* November 2020;49(D1):D884–D891. 10.1093/nar/gkaa942 PMC7778975 33137190

[98] Childers JW Back AL Tulsky JA : REMAP: A framework for goals of care conversations. *J. Oncol. Pract.* October 2017;13(10):e844–e850. 10.1200/JOP.2016.018796 28445100

[99] Zhao H Sun Z Wang J : CrossMap: a versatile tool for coordinate conversion between genome assemblies. *Bioinformatics.* December 2013;30(7):1006–1007. 10.1093/bioinformatics/btt730 24351709 PMC3967108

[100] Turner SD : qqman: an r package for visualizing GWAS results using q-q and manhattan plots. *J. Open Source Soft.* May 2018;3(25):731. 10.21105/joss.00731

[101] Leeuw CA de Mooij JM Heskes T : MAGMA: Generalized gene-set analysis of GWAS data. *PLoS Comput. Biol.* April 2015;11(4):e1004219. 10.1371/journal.pcbi.1004219 25885710 PMC4401657

[102] Marbach D Lamparter D Quon G : Tissue-specific regulatory circuits reveal variable modular perturbations across complex diseases. *Nat. Meth.* March 2016;13(4):366–370. 10.1038/nmeth.3799 26950747 PMC4967716

