# Supplementary material for: Performing post-genome-wide association study analysis: overview, challenges and recommendations
Source: F1000Res. 2021 Oct 4;10:1002. [Version 1] doi: 10.12688/f1000research.53962.1 (PMC8847724; doi:10.12688/f1000research.53962.1)
Supplement: Peer review history

# Peer review: Reviewer response for version 1

**DOI:** 10.5256/f1000research.57399.r122325

**Published:** 2022-02-15

**Authors:** Yasuhiro Sato

**Affiliations:** 1 University of Zurich, Zürich, Switzerland

This review paper entitled “Performing post-genome-wide association study analysis: overview, challenges and recommendations” provides a practical guide to post-GWAS analyses in human complex traits and diseases. To be honest, I am not a human geneticist, but find this paper helpful for a non-human geneticist to overview a wide variety of post-GWAS analyses. While the principles are not so deeply discussed, the example code helps us use software and interpret its result. I have a few concerns on the step 3 of downstream analyses.

**Major comments:**

1. Usage of the heritability estimation
  The authors mention “…, the ability of GWAS to detect a significant genomic position depends on heritability on phenotype, minor allele frequency…” (Introduction) and “Higher values of heritability indicate that the phenotype is explained best by the genotype, i.e., set of SNPs” (Chip heritability section). This understanding seems correct, but I am wondering why the heritability estimation should be conducted ‘after’ GWAS. If the SNP heritability is a good proxy to know the extent to which a target trait is genetically controlled, we should perform the heritability estimation ‘before’ GWAS analysis. Some web-based GWAS pipelines provide SNP heritability before performing association mapping (e.g., GWA-Portal: Seren 2018 Methods Mol Biol).
2. Gene-set analysis
  Because a gene-set or gene ontology enrichment analysis is widely performed in omics analyses (e.g., RNA-Seq), further introduction to the gene-set analysis would gain a value of this manuscript. As far as I know, due to the LD among SNPs, gene-set enrichment analyses in GWAS are not so straightforward as those in RNA-Seq and the other omics analyses. For example, Gowinda is designed for an unbiased gene-set enrichment analysis for GWAS (Kofler & Schlötterer 2012 Bioinformatics).

**Minor Comments:**

1. Page 4 of 20 “Plink”, Page 6 of 20, and elsewhere: PLINK is described as “PLINK” in some lines but also as “Plink” in the other lines. The capital letters would be better.
2. Page 5 of 20: The equation of the Bayesian rule is incorrect. *P*( *M*) should be a numerator.
3. Page 13 of 20 “Statistics for colocalization studies”: This subsection is not informative as it consists of only a single sentence.
4. Page 13 of 20 “Resources for colocalization studies”: This seems a list of papers and thus repetitive of References. Please explain the points of these papers, otherwise this list is unnecessary.
5. Page 15 of 20: Letters in Figure 1 are too small to read.
6. As this review focuses on tools rather than theory, it would be great if the authors could deposit their tutorial (incl. codes and example data) on some open repository.

Is the rationale for developing the new method (or application) clearly explained?

Yes

Is the description of the method technically sound?

Yes

Are the conclusions about the method and its performance adequately supported by the findings presented in the article?

Yes

If any results are presented, are all the source data underlying the results available to ensure full reproducibility?

No source data required

Are sufficient details provided to allow replication of the method development and its use by others?

Yes

Reviewer Expertise:

plant ecology and genetics

I confirm that I have read this submission and believe that I have an appropriate level of expertise to confirm that it is of an acceptable scientific standard.

## References

[1] : GWA-Portal: Genome-Wide Association Studies Made Easy. *Methods Mol Biol* .2018;1761: 10.1007/978-1-4939-7747-5_22 303-319 10.1007/978-1-4939-7747-5_22 29525966

[2] : Gowinda: unbiased analysis of gene set enrichment for genome-wide association studies. *Bioinformatics* .2012;28(15) : 10.1093/bioinformatics/bts315 2084-5 10.1093/bioinformatics/bts315 22635606PMC3400962

# Peer review: Reviewer response for version 1

**DOI:** 10.5256/f1000research.57399.r98101

**Published:** 2021-11-15

**Authors:** Zhe Zhang

**Affiliations:** 1 Guangdong Laboratory of Lingnan Modern Agriculture/Guangdong Provincial Key Lab of Agro-Animal Genomics and Molecular Breeding, College of Animal Science, South China Agricultural University, Guangzhou, China

The authors of the manuscript entitled “Performing post-genome-wide association study analysis: overview, challenges and recommendations” give a detailed overview to the methods and tools of post GWAS analysis. The principles, key factors, tools, resources, suggestions and codes were provided to facilitate researchers who need to conduct post GWAS analysis. Overall, the manuscript is well organized and well written. It would be helpful for all target readers. There are few of my concerns that should be addressed by the authors.

 Minor comments:

 “Introduction” section: the authors divided common pGWAS analysis approaches into “the following three approaches ...”. Actually, nine approaches were mentioned in the main text. Hence, rewording of this paragraph is suggested.  

 “Bayesian methods: framework” section: the equation for Bayes’ rule is not correct.

 “Colocalization analysis” section: Providing more information is suggested, since the content in the current version of this section is a bit too simple. Such as “Several parametric and non-parametric statistics…”.

Is the rationale for developing the new method (or application) clearly explained?

Yes

Is the description of the method technically sound?

Yes

Are the conclusions about the method and its performance adequately supported by the findings presented in the article?

Yes

If any results are presented, are all the source data underlying the results available to ensure full reproducibility?

No source data required

Are sufficient details provided to allow replication of the method development and its use by others?

Yes

Reviewer Expertise:

statistical genomics, animal breeding

I confirm that I have read this submission and believe that I have an appropriate level of expertise to confirm that it is of an acceptable scientific standard.
